# Elevated pyrimidine dimer formation at distinct genomic bases underlies promoter mutation hotspots in UV-exposed cancers

**DOI:** 10.1371/journal.pgen.1007849

**Published:** 2018-12-26

**Authors:** Kerryn Elliott, Martin Boström, Stefan Filges, Markus Lindberg, Jimmy Van den Eynden, Anders Ståhlberg, Anders R. Clausen, Erik Larsson

**Affiliations:** 1 Department of Medical Biochemistry and Cell Biology, Institute of Biomedicine, The Sahlgrenska Academy, University of Gothenburg, Gothenburg, Sweden; 2 Sahlgrenska Cancer Center, Department of Pathology and Genetics, Institute of Biomedicine, Sahlgrenska Academy at University of Gothenburg, Gothenburg, Sweden; 3 Wallenberg Centre for Molecular and Translational Medicine, University of Gothenburg, Sweden; 4 Department of Clinical Pathology and Genetics, Sahlgrenska University Hospital, Gothenburg, Sweden; National Institute of Environmental Health Sciences, UNITED STATES

## Abstract

Sequencing of whole cancer genomes has revealed an abundance of recurrent mutations in gene-regulatory promoter regions, in particular in melanoma where strong mutation hotspots are observed adjacent to ETS-family transcription factor (TF) binding sites. While sometimes interpreted as functional driver events, these mutations are commonly believed to be due to locally inhibited DNA repair. Here, we first show that low-dose UV light induces mutations preferably at a known ETS promoter hotspot in cultured cells even in the absence of global or transcription-coupled nucleotide excision repair (NER). Further, by genome-wide mapping of cyclobutane pyrimidine dimers (CPDs) shortly after UV exposure and thus before DNA repair, we find that ETS-related mutation hotspots exhibit strong increases in CPD formation efficacy in a manner consistent with tumor mutation data at the single-base level. Analysis of a large whole genome cohort illustrates the widespread contribution of this effect to recurrent mutations in melanoma. While inhibited NER underlies a general increase in somatic mutation burden in regulatory elements including ETS sites, our data supports that elevated DNA damage formation at specific genomic bases is at the core of the prominent promoter mutation hotspots seen in skin cancers, thus explaining a key phenomenon in whole-genome cancer analyses.

## Author summary

Cancer is caused by somatic mutations that typically occur in protein-coding genes. However, the advent of whole genome sequencing has made it possible to venture beyond protein-coding DNA in search of non-coding mutations with putative cancer driver roles. Indeed, recent studies, in particular in skin cancers, describe individual positions in gene regulatory regions (promoters) that are recurrently mutated in many independent patients, suggestive of a contribution to carcinogenesis. In this paper, we show that recurrent promoter mutations arise at these sites due to an exceptional propensity to form UV-induced DNA damage lesions (pyrimidine dimers) at specific transcription factor binding sites. The effect is present in cellular DNA but not in naked acellular DNA, meaning that the sites need to be occupied by their transcription factor partners in order to induce favorable conditions for DNA damage formation. This explains an important confounding phenomenon in whole cancer genome analyses, and has implications for the interpretation of recurrent somatic mutation patterns in non-coding DNA.

## Introduction

Whole genome analysis of cancer genomes has the potential to reveal non-coding somatic mutations that drive tumor development, but it remains a major challenge to separate these events from non-functional passengers. The main principle for identifying drivers is recurrence across independent tumors, suggestive of positive selection, which led to the recent identification of frequent oncogenic mutations in the promoter of telomere reverse transcriptase (*TERT*) that can activate its transcription [1, 2]. However, mutation rates vary across the genome, and local elevations may give rise to “false” recurrent events that can be misinterpreted as signals of positive selection. While known covariates of mutation rate, such as replication timing and chromatin organization [3, 4], transcriptional activity [5] and local trinucleotide context [6], can be accounted for to improve interpretation [7], the non-coding genome may be particularly challenging. Mutational fidelity may be generally reduced in this vast and relatively unexplored space, as indicated by the presence of mechanisms directing DNA repair specifically to gene regions [8, 9], and yet-unexplained mutational phenomena may be at play.

Indeed, recent studies have described a remarkable abundance of recurrent promoter mutations in melanoma and other skin cancers, often noted to overlap with sequences matching the recognition element of ETS family transcription factors (TFs) [10–16]. Strikingly, a large proportion of frequently recurring promoter mutations in melanoma occur at distinct cytosines one or two bases upstream of TTCCG elements bound by ETS factors as indicated by ChIP-seq, within a few hundred bases upstream of a transcription start site [17]. While often interpreted as driver events, we recently showed that these sites exhibit highly elevated vulnerability to UV mutagenesis, as evidenced by their rapid induction following low-dose UV light exposure in cultured cells [17]. The effect has often been attributed to locally impaired nucleotide excision repair (NER) caused by binding of ETS TFs [16, 18, 19]. However, our analysis of skin tumors lacking global NER (*XPC* -/-) contradicted this model [17] and the mechanism remains unclear. An understanding of this phenomenon, which may underlie a large part of all non-coding recurrent events in human tumors beyond *TERT* [10, 12, 16], would resolve a key question that continues to confound whole cancer genome analyses.

Here, through analysis of 221 whole tumor genomes, we first demonstrate the widespread impact of TTCCG-related mutagenesis on the mutational landscape of melanoma. Moreover, through UV exposure of a panel of repair-deficient human cell lines, we rule out inhibited DNA repair as core mechanism. Finally, we generate the highest resolution map of UV-induced cyclobutane pyrimidine dimers (CPDs) in the human genome to date, which provides clear evidence that ETS-related promoter hotspots are associated with strong local elevations in the efficacy of UV lesion formation at specific genomic bases.

## Results

### Widespread contribution from TTCCG-related sites to recurrent non-coding mutations in 221 melanoma whole genomes

To assess the impact of TTCCG-related mutagenesis on the landscape of recurrent mutations in melanoma in a more sensitive way than previously possible, we assembled a cohort of 221 melanomas characterized by whole genome sequencing by TCGA and ICGC [20, 21]. These heavily mutated tumors averaged 110k somatic single nucleotide variants (SNVs) per sample, expectedly dominated by C>T transitions and a mutational signature characteristic of mutagenesis by UV light through formation of pyrimidine dimers (S1 Fig).

**Fig 1:**
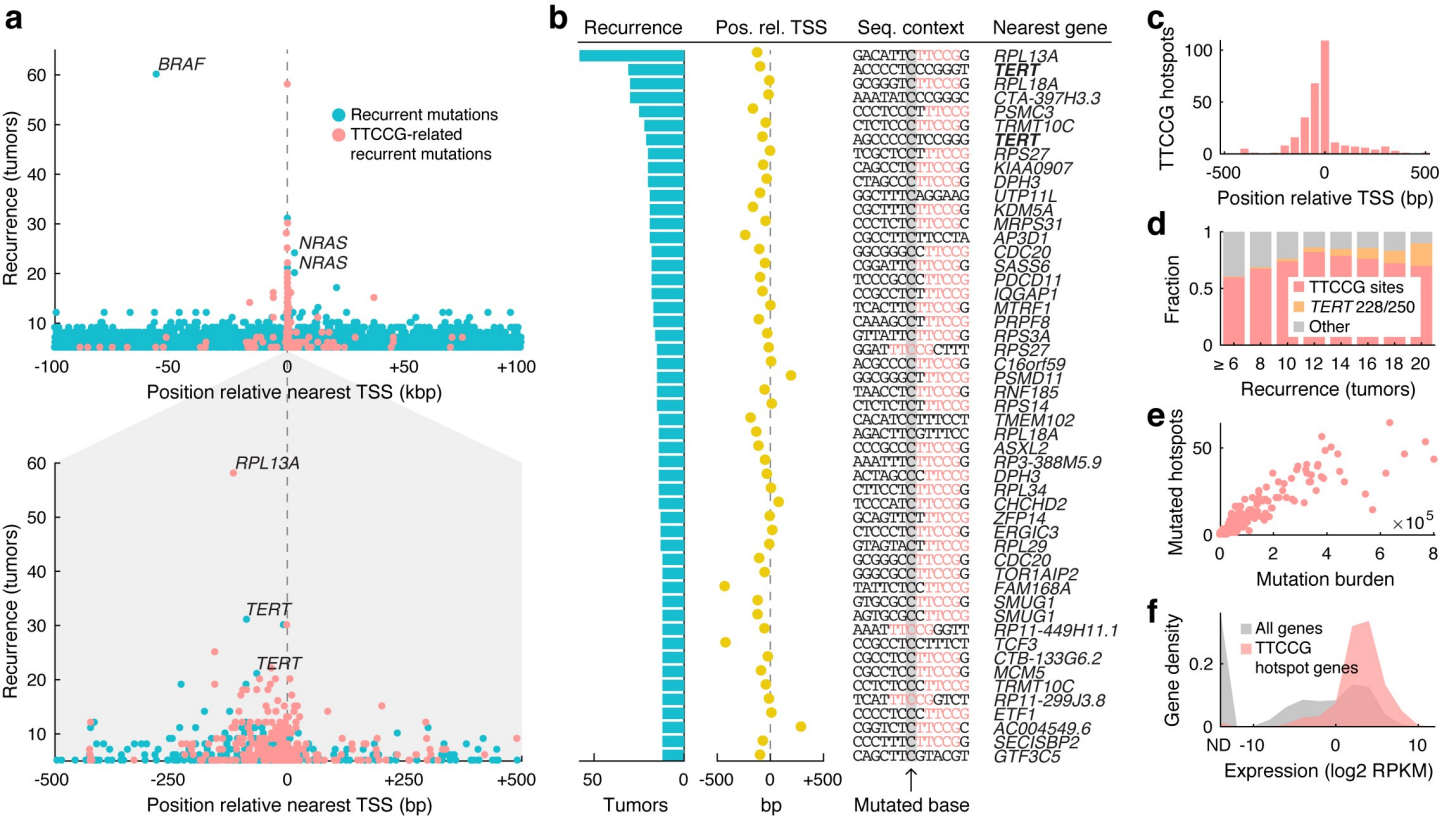
Widespread contribution from TTCCG-related sites to recurrent non-coding mutations in 221 whole melanoma genomes. (**a**) Highly recurrent somatic mutations (individual genomic bases) aggregate near annotated transcription start sites (TSS) and typically colocalize with TTCCG elements. Recurrent sites having a TTCCG element within a +/-10 bp context on the mutated (pyrimidine) strand are indicated (red). The distance to the nearest TSS (x-axis) is adjusted for transcriptional orientation (upstream positioning of *BRAF* V600E mutations is explained by their relative proximity to the neighboring *NDUFB2* promoter). Bottom panel: +/-500 bp close-up around the TSS. (**b**) Top 51 recurrent promoter sites (+/-500 bp), all mutated in ≥12/221 tumors (>5%). Degree of recurrence, position relative to TSS, sequence context with TTCCG highlighted in red, and nearest gene are indicated. (**c**) Positional distribution of TTCCG-related mutation hotspots near TSSs, based on 291 promoter sites recurrent in ≥5 tumors. (**d**) Proportion of recurrent promoter mutations (+/- 500 bp) that are TTCCG-related (red), *TERT* activating mutations (C228T/C250T; orange) or other (gray), as a function of recurrence. (**e**) Number of mutated TTCCG promoter hotspot sites per tumor, out of 291 in total as defined above, plotted against the whole-genome mutational burden across 221 melanomas. (**f**) TTCCG-related promoter hotspots arise preferably near highly expressed genes. 241 genes hosting 291 sites as defined above were considered. Expression levels were based on the median RPKM value across a subset of 38 TCGA melanomas with available RNA-seq. ND, not detected.

Notably, despite the genome-wide scope, nearly all highly recurrent mutations were found near annotated transcription start sites (TSSs) (Fig 1a). For example, of the 22 most recurrent individual bases (mutated in ≥18 patients), four were known drivers (*BRAF*, *NRAS* or *TERT* promoter mutations) while the rest were at most 524 bp away from a known TSS. Further, the vast majority of highly recurrent promoter sites were found in conjunction with TTCCG sequences (Fig 1a and 1b), indicating a widespread influence from ETS elements to the mutational landscape of melanoma. Analysis of TTCCG-related recurrent mutations in relation to enhancers further supported that the effect is largely restricted to promoters (S2 Fig).

Of 51 recurrent promoter mutations (+/- 500 bp from TSS) mutated in ≥ 12 tumors, 42 (82%) had a TTCCG element in the immediate (+/- 10 bp) sequence context, rising to 86% after excluding the known *TERT* C228T and C250T promoter mutations (Fig 1b and S1 Table) [1, 2]. Most were within 200 bp upstream of a known TSS, as expected for functional ETS elements (Fig 1b and 1c) [22]. Among the few remaining sites, two (upstream of *AP3D1* and *TMEM102*) were instead flanked by TTCCT sequences likewise matching the ETS recognition motif (Fig 1b) [22] and the numbers are thus conservative. The fraction TTCCG-related sites increased as a function of recurrence, from 291/550 promoter sites (53%) at *n* ≥ 5 to 7/8 (88%) at *n* ≥ 20, excluding the known *TERT* sites (Fig 1d). For comparison, only 0.60% of C>T mutations in the dataset exhibited TTCCG patterns, underscoring their massive enrichment in recurrent positions.

As noted previously [17], there was a strong correlation between the number of mutated TTCCG hotspot sites and the total mutational burden in each tumor, compatible with these sites being passive passengers (Spearman’s *r* = 0.94, *P* = 1.5e-106; Fig 1e). Also confirming earlier observations, the TTCCG-related promoter hotspots were found preferably near highly expressed genes, as expected under a model where interaction with an ETS TF rather than sequence-intrinsic properties are responsible for elevated mutation rates in these sites (Fig 1f). Taken together, these analyses clearly demonstrate that ETS-related mutations account for nearly all highly recurrent non-coding hotspots genome-wide in melanoma, as well as hundreds of less recurrent sites not detectable in previous analyses based on smaller cohorts.

### The *RPL13A* TTCCG hotspot shows elevated sensitivity to UV mutagenesis *in vitro* in the absence of repair

Recent studies have shown that NER, the main DNA repair pathway for UV damage, is attenuated in TF binding sites, leading to elevated somatic mutation rates [18, 19]. While plausible as a mechanism for ETS-related mutation hotspots [16], we recently showed that TTCCG elements were associated with elevated mutation rates also in cutaneous squamous cell carcinomas (cSCCs) lacking global NER (*XPC* -/-) [17]. We also established that mutations can be easily induced in TTCCG hotspot sites in cell culture by UV light, thus recreating *in vitro* the process leading to recurrent mutations in tumors [17]. We decided to use the *RPL13A* -116 bp hotspot site, notably more frequently mutated (58/221 tumors) than both canonical *TERT* sites and on par with *BRAF* V600E at 60/221 (Fig 1a and 1b), as a model to further investigate a possible role for impaired NER.

**Fig 2:**
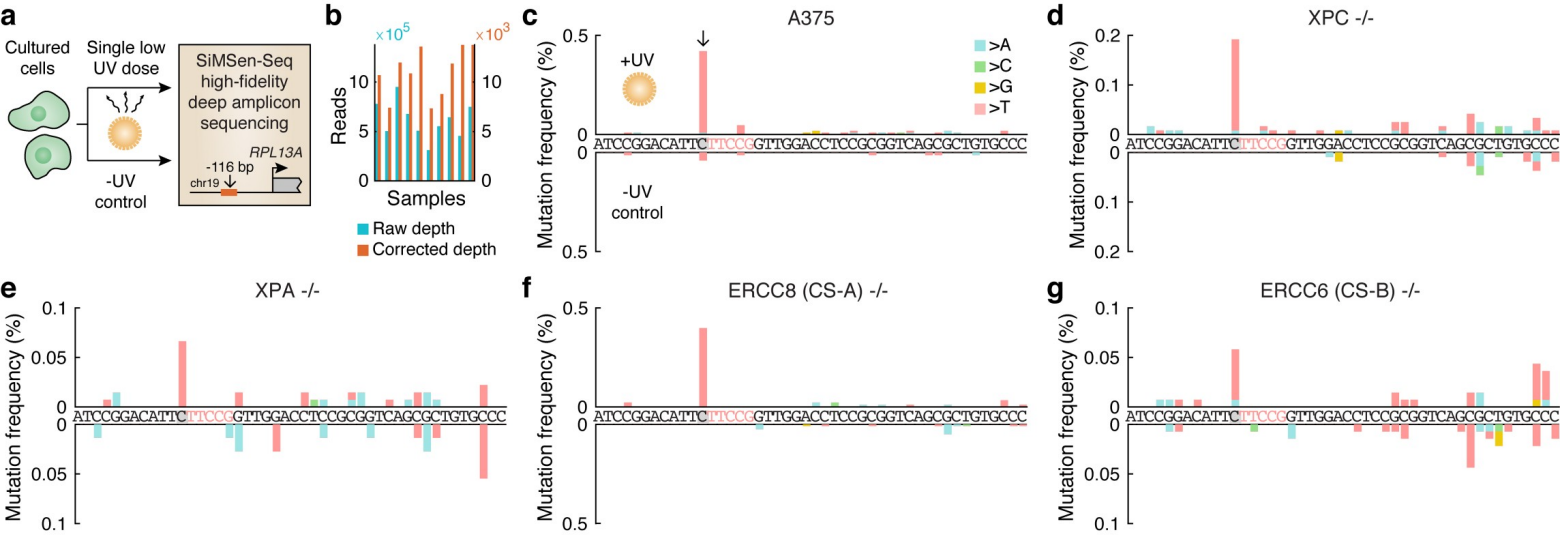
UV exposure of cultured cells induces mutations preferably at the *RPL13A* TTCCG hotspot site independently of repair. (**a**) Cultured human cells, either A375 melanoma cells or fibroblasts with NER deficiencies, were subjected to a single UVB dose (20 J/m^2^) during approx. 2 seconds. Following recovery, cellular DNA was subsequently assayed for subclonal mutation in the *RPL13A* -116 bp TTCCG promoter hotspot site (see Fig 1a and 1b, top row) using SiMSen-Seq error-corrected amplicon sequencing [23]. Non-UV-treated sample were included as controls. (**b**) 10 samples were sequenced at 311k to 950k reads each, resulting in 7.3k to 13.8k error-corrected reads at ≥10x oversampling. (**c**) Subclonal mutations in a 46 bp amplicon window encompassing the *RPL13A* -116 bp hotspot in A375 melanoma cells. The hotspot site and TTCCG element are indicated in gray/red, respectively. Positive axis, UV-treated sample; negative axis, no UV control. (**d-g**) As panel **c** but showing results from *XPC* -/- (lacking global NER), *XPA* -/- (lacking global and transcription-coupled NER), *ERCC8* -/- and *ERCC6* -/- (lacking transcription-coupled NER) mutant fibroblasts.

To this end, we UV-exposed A375 cells with intact NER as well as fibroblasts with homozygous mutations in four key DNA repair components: *XPC*, required for global NER, *ERCC8* (*CSA*) and *ERCC6* (*CSB*), required for transcription coupled NER (TC-NER), and *XPA* which is required for lesion verification in both global and TC-NER (S3 Fig). Correct genetic identity and complete homozygosity for the mutant allele was confirmed by whole-genome sequencing of all four mutant cell lines (S2 Table). Even limited UV exposure led to high cell mortality in the mutant cell lines, forcing us to limit the exposure to a single low dose of UVB (20 J/m^2^) during approximately two seconds followed by three weeks of recovery, after which cells were assessed for *RPL13A* promoter mutations using error-corrected amplicon sequencing (Fig 2a) [23]. Between 7,332 and 13,774 error-corrected reads at ≥10x oversampling were obtained for each of 10 different libraries (Fig 2b).

Strikingly, even at this miniscule dose, subclonal somatic mutations appeared preferably at the known hotspot site in A375 cells (Fig 2c) as well as in all of the mutant cell lines (Fig 2e–2g), despite abundant possibilities for UV lesion formation in flanking assayed positions. As expected, absolute mutation frequencies were low, less than 0.5% in all samples, bringing us close to the detection limit in some samples as indicated by noise in the untreated controls (Fig 2d, 2e and 2g). In combination with earlier data from tumors lacking global NER [17] and the fact that the mutations are almost exclusively positioned upstream of TSSs where TC-NER should not be active (Fig 1b and 1c), these results argue against impaired global NER as well as TC-NER as the basic mechanism behind TTCCG hotspot formation.

### High-resolution mapping of CPDs across the human genome

**Fig 3:**
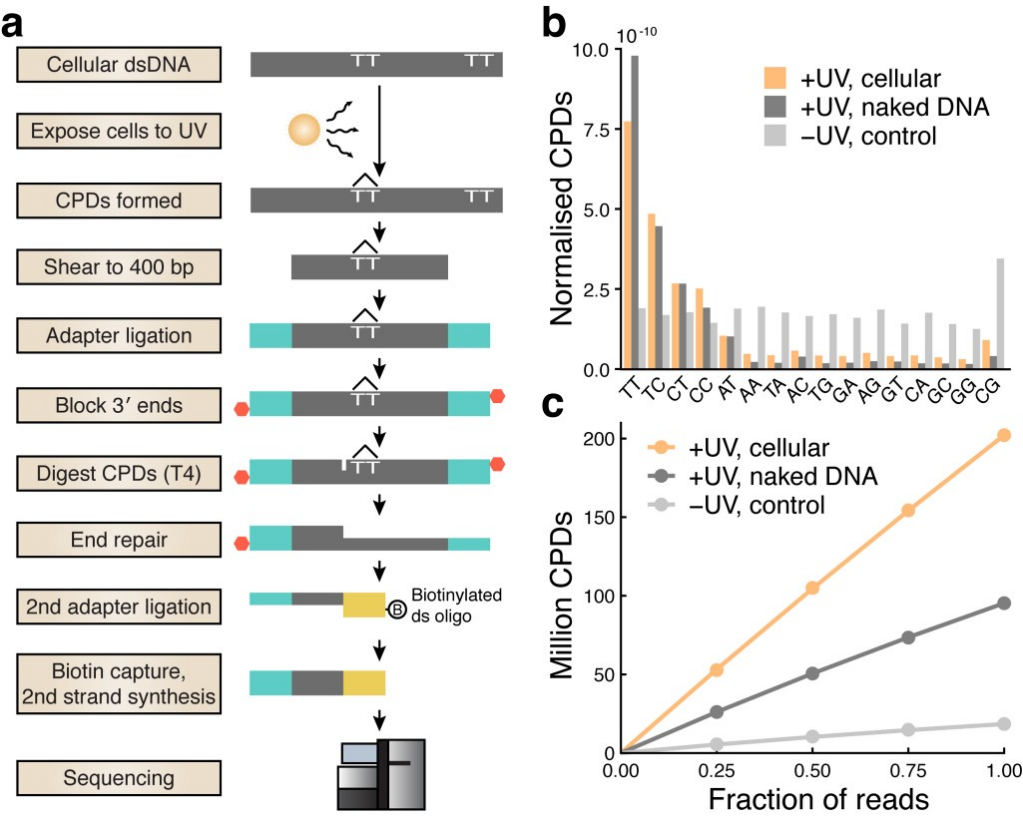
High-coverage mapping of UV-induced cyclobutane pyrimidine dimers across the human genome. (**a**) Schematic of the experimental protocol. (**b**) Distribution of dinucleotides at which CPDs were detected, showing an expected preference for dipyrimidines. Counts from cellular, naked (acellular) and no-UV-control samples were normalized with respect to genomic dinucleotide counts as well as sequencing depth. (**c**) The number of detected CPDs in each library following removal of PCR duplicates shown at full depth, as well as based on subsampled data (25, 50 and 75%) to simulate lower sequencing depth.

It was established decades ago that DNA conformational changes induced by interactions with proteins can alter conditions for UV damage formation [24, 25], which prompted us to investigate whether ETS-related promoter hotspots may arise due to locally favorable conditions for UV lesion formation. For this, we adapted a protocol first established in yeast using IonTorrent sequencing [26] to the Illumina platform (Fig 3a), to generate a genome-wide map of CPDs in A375 human melanoma cells immediately following UV exposure, before DNA repair processes have had a chance to act.

CPDs were preferably detected at TT, TC, CT and CC dinucleotides as expected (Fig 3b). An elevation at AT dinucleotides was consistent with an earlier report where this was attributed primarily to AT[T/C] sites, suggesting a contribution from CPDs at flanking dipyrimidines [26]. By comparing with median detection frequencies at non-dipyrimidines, we estimated the false positive rate for CPDs at dipyrimidines to vary from 5.5% for TT up to 17.0% for CC. The number of detected CPDs after removal of PCR duplicates scaled nearly linearly with simulated sequencing depth, indicating favorable random representation of CPDs (Fig 3c). A total of 202.1 million CPDs were mapped to dipyrimidines throughout the cellular genome (Fig 3c), constituting the highest resolution CPD map to date to our knowledge. Additionally, 95.3 million CPDs were mapped in UV-treated naked (acellular) A375 DNA lacking interacting proteins, while a non-UV-treated control, which expectedly yielded limited material, produced 18.5 million CPDs (Fig 3c).

### CPD formation spikes at TTCCG-related promoter mutation hotspots

**Fig 4:**
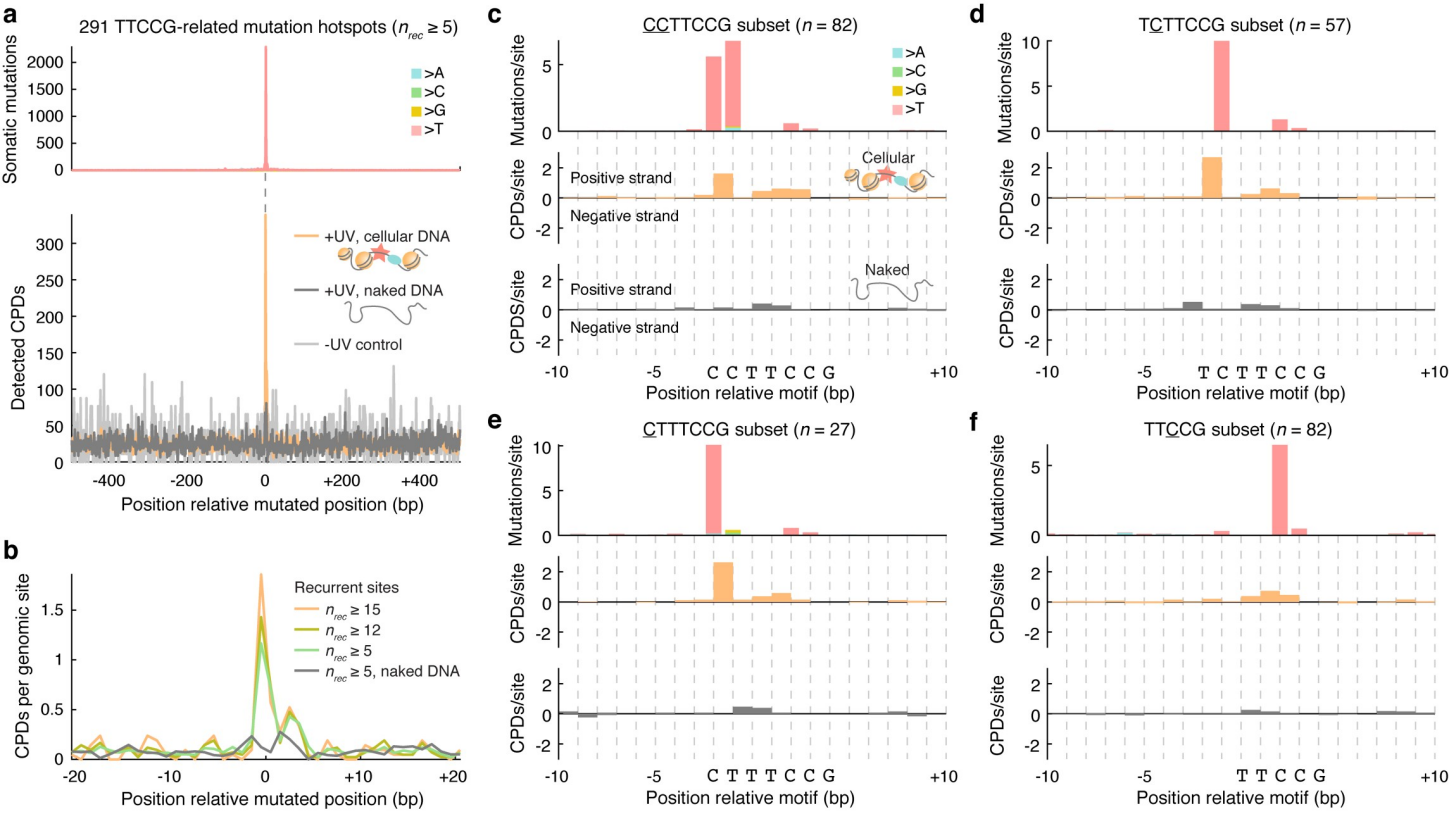
CPD formation spikes at TTCCG-related promoter mutation hotspots. (**a**) 291 recurrently mutated (*n* ≥ 5/221 melanomas) genomic promoter sites (+/-500 bp from nearest TSS), as defined and illustrated in Fig 1a and 1b, were aligned centered on the mutated base (in each case considering the pyrimidine-containing strand, i.e. C, for the mutated base in the reference genome). The top and bottom panels show mutation and CPD formation density, respectively, in a +/- 500 bp window centered on the mutated base. Naked DNA (dark grey) and no-UV control (light grey) whole-genome CPD counts were normalized to be comparable to the cellular DNA data (orange). (**b**) Close-up view (+/- 20 bp) showing CPD density for different subsets of the 291 sites, defined by the degree of mutation recurrence, revealing that more prominent melanoma mutation hotspots show stronger CPD formation signals. (**c-f**) Detailed view of CPD formation patterns in TTCCG promoter mutation hotspot sites after subcategorization into four main groups based on sequence and mutated position (mutated base indicated by underscore, with CCTTCCG sites typically showing recurrent mutations at both 5ʹ cytosines). Mutated genomic regions were aligned centered at the start of the motif while removing redundant (non-unique) genomic loci. CPD frequencies are shown separately for the positive and negative strands, for both cellular (orange) and naked (grey) DNA. Mutation and CPD formation frequencies were normalized by the number of hotspot sites in each alignment, following depth-normalization as described in panel **a**.

We next investigated CPD formation patterns at TTCCG mutation hotspot positions identified above in melanoma (Fig 1a and 1b). 291 recurrently mutated (*n* ≥ 5/221 melanomas) TTCCG promoter sites (+/-500 bp from TSS) were aligned centered on the mutated base such that CPD density in these regions could be determined. This revealed a striking peak in CPD formation that coincided with the hotspots, which was largely absent in naked DNA lacking bound proteins or in non-UV control DNA (Fig 4a). Additionally, more recurrently mutated sites showed a stronger CPD signal, compatible with increased CPD formation being the key mechanism (Fig 4b).

For a more detailed understanding, we subcategorized the 291 melanoma ETS hotspot sites into four main groups based on sequence and mutated position. The strongest mutation hotspots, such as *RPL13A* and *DPH3*, typically occurred at cytosines one or two bases upstream of the TTCCG element (Fig 1b and S1 Table), which notably is outside of the core motif and therefore not expected to disrupt binding [22]. In CCTTCCG sites (*n* = 82 unique loci), recurrent C>T transitions would typically appear at both 5ʹ cytosines (underscored) independently or, less frequently, as CC>TT double nucleotide substitutions. Aggregated CPD density over these sites, centered on the motif, revealed a strong peak bridging these two bases, which notably was absent in naked DNA (Fig 4c). Thus, when the TF site is occupied, CPDs form efficiently between the two pyrimidines, leading to C>T mutations at either base although with a preference for the second position, in agreement with established models for UV mutagenesis [27]. The same pattern of strongly elevated CPD formation in cellular, but not naked, DNA was observed between the same positions in TCTTCCG and CTTTCCG sites (*n* = 57 and 27, respectively), with C>T mutations expectedly forming only at the first or second pyrimidine depending on the position of the cytosine (Fig 4d and 4e).

Many of the less recurrent bases in melanoma were often found at the first middle cytosine of a TTCCG motif (S1 Table). Interestingly, a large fraction of these sites lacked a dipyrimidine at the two key positions identified above thus prohibiting CPD formation there, with ACTTCCG being the most common pattern (44/82 sites), which indeed matches the *in vivo* ETS consensus sequence [22]. Compatible with the mutation data, the strongest CPD peak was observed at the middle TC dinucleotide, and in agreement with the lower mutation recurrence, this signal was weaker compared to the other site types (Fig 4f). Of note, elevated CPD formation between these bases could also be clearly seen in the other site categories (Fig 4c–4e). Taken together, these analyses based on genome-wide CPD mapping provide strong evidence that locally elevated CPD formation efficacy shapes the formation of mutation hotspots at ETS binding sites.

### Overall elevated mutation rate in regulatory regions is not due to increased CPD formation

**Fig 5:**
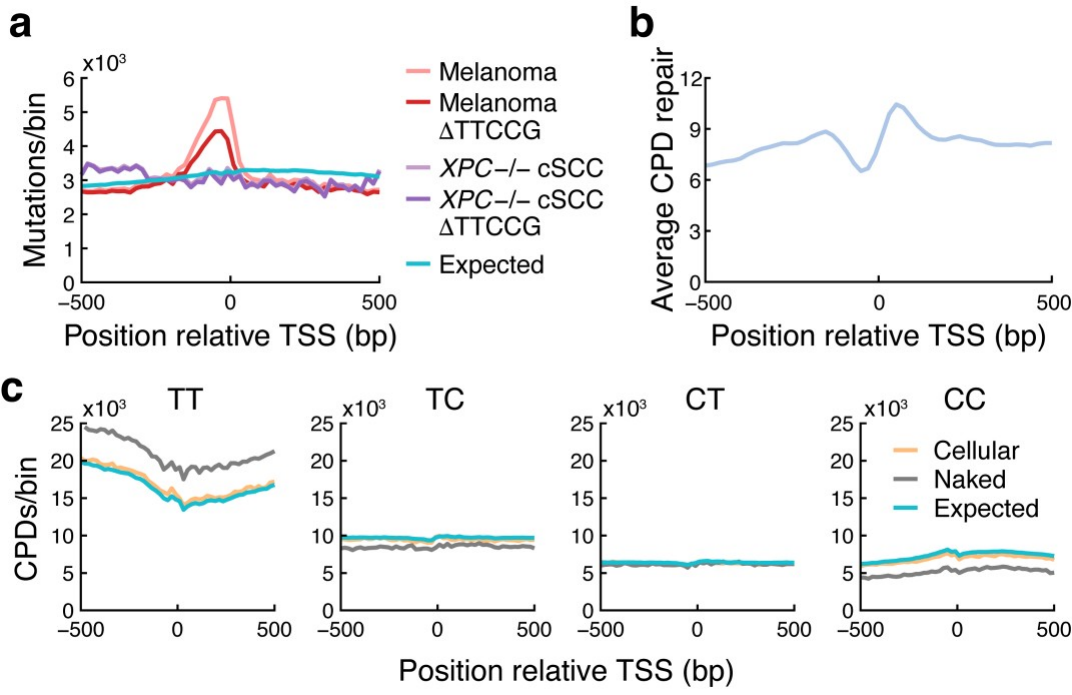
Overall elevated mutation rate in regulatory regions is not due to increased CPD formation. (**a**) Somatic C>T mutation density around annotated TSSs in melanomas and *XPC* -/- cSCCs lacking global NER, per 20 bp genomic bin, aggregated over 33,456 coding genes and lncRNAs in the GENCODE v19 annotation. Densities after subtracting mutations having a TTCCG element in the immediate (+/- 10 bp) sequence context are also shown (ΔTTCCG). Expected mutation counts were determined by generating an equal number of mutations using observed trinucleotide mutational signatures in the melanoma samples. cSCC mutation counts were normalized to be comparable to melanoma. (**b**) Average NER activity as determined by XR-Seq [28] in TSS regions. (**c**) Observed CPD counts in TSS regions (20 bp bins) in cellular and naked DNA, presented per CPD-forming dinucleotide. Naked DNA counts were normalized to be comparable to the cellular DNA data. Expected counts were determined based on dinucleotide counts in the analyzed regions.

Earlier studies have described a general increase in mutation rate in promoter regions, attributed to reduced NER activity at sites of TF binding including ETS sites [18, 19]. To investigate a possible contribution from increased CPD formation to this pattern, we first determined the overall mutation rate in melanoma near TSSs, which confirmed a sharp increase in upstream regions that coincided with reduced NER as determined by XR-Seq (Fig 5a and 5b) [28]. Further confirming earlier data [19], this increase was abrogated in *XPC* -/- cSCCs lacking global NER, arguing against a major contribution from increased CPD formation (Fig 5a). Consistent with this, CPDs were found to form at near-expected frequencies when aggregated over these regions (Fig 5c). Interestingly, subtraction of TTCCG-related mutations revealed that these constitute a large proportion of promoter mutations in melanomas, but not in *XPC* -/- cSCCs, supporting a notable contribution from inhibited NER in ETS sites to the overall burden increase in promoters (Fig 5a).

While elevated UV-induced DNA damage is important in the formation of ETS-related recurrent mutation hotspots, we conclude that this effect has negligible impact on the general increase in mutation burden in regulatory regions. This is instead explained by repair inhibition including a prominent contribution from impaired NER in ETS sites, which can likely further add to elevated mutation frequencies at recurrent hotspot positions.

## Discussion

Proper analysis of recurrent non-coding mutations requires an understanding of how mutations arise and distribute across the genome in the absence of selective pressures. Here, we provide a mechanistic explanation for the passive emergence of recurrent mutations at specific positions in TTCCG/ETS sites in tumors in response to UV light, and also demonstrate the massive impact of such mutations on the mutational landscape of melanoma using a large whole genome cohort.

Mutations at -116 bp in the *RPL13A* promoter were used here as a model to study mutation formation at ETS hotspot sites *in vitro* in repair-deficient cell lines, which ruled out inhibited repair as sole mechanism. Of note, this site is more recurrently mutated than the individual *TERT* C228T/C250T sites and nearly as frequent as chr7:140453136 mutations (hg19) pertaining to *BRAF* V600E, thus representing the second most common mutation in melanoma and likely other skin cancers. Notably, mutations were detectable at this site in cultured cells following a UVB dose of 20 J/m^2^ UVB, equivalent to about 1/200th of the monthly absorbed UVB dose in July in Northern Europe [29]. This underscores the extreme UV sensitivity of ETS hotspots and explains their high recurrence in tumors.

Genome-wide mapping of CPDs revealed that TTCCG-related mutation hotspots exhibit highly efficient CPD formation at the two bases immediately 5ʹ of the core TTCC ETS motif. The effect was lost in naked acellular DNA, showing that structural conditions for elevated CPD formation are induced when the TF binding site is in its protein-bound state. Interestingly, most functional ETS sites are expected to lack pyrimidines in the two key positions [22] thus prohibiting pyrimidine dimer formation, and conditions for forming a strong mutation hotspot are therefore only met in a subset of sites with CC, TC or CT preceding the TTCCG element. Additionally, CPDs form at lower but still elevated frequency at the middle TTCCG bases, consistent with weaker recurrence for mutations in these positions. CPD and cancer genomic data are thus in strong agreement, providing a credible mechanism for the formation of ETS-related mutations hotspots in UV-exposed cancers.

As demonstrated here, frequent mutations at ETS-site hotspots are expected for purely biochemical reasons in UV-exposed cancers. Consequently, several observations are compatible with passenger roles for these mutations: The most recurrent sites arise at cytosines outside of the core TTCC ETS recognition element [22] where they are not expected to disrupt ETS binding. While mutations in the middle of the motif, common among the less frequent hotspots, should disrupt binding, ETS factors tend to be oncogenes that are activated in cancer [30], and it can be noted that *TERT* promoter mutations instead enable ETS binding through formation of TTCC elements [1, 2]. The mutations tend to arise near highly expressed housekeeping genes rather than cancer-related genes, and the particular set of sites that are mutated varies inconsistently in-between tumors. Moreover, as would be expected in the absence of selection and in contrast to known driver mutations [17], the number of mutated ETS sites in a tumor is strongly determined by mutational burden.

Our results complement a recent study by Mao *et al*. [31], which was published during the preparation of this manuscript. This study likewise determined CPD formation patterns in ETS binding sites using whole genome CPD mapping obtaining results that are in full agreement with ours, and additionally proposed a structural basis based on available crystallography data for increased CPD formation at the center TC dinucleotide in the ETS-DNA complex, which was demonstrated to promote CPD formation also *in vitro*. Thus, data on CPD formation patterns from two independent studies, in combination with our data showing a sharply elevated mutation rate at the *RPL13A* TTCCG hotspot site *in vitro* in the absence of NER, support that base-specific elevations in CPD formation efficacy forms the foundation for prominent promoter mutation hotspots in skin cancers. At the same time, inhibited DNA repair explains a general increase in mutation burden in regulatory elements including ETS sites, which could act synergistically to further amplify elevated mutation rates at ETS-related hotspots. Future studies may want to better quantify the relative contributions of these effects, as well as define the exact subset of ETS factors or other proteins that interact with DNA at TTCCG-related mutation hotspot sites.

## Materials and methods

### Whole genome mutation analyses

Whole genome somatic mutation calls from the Australian Melanoma Genome Project (AMGP) cohort [20] were downloaded from the International Cancer Genome Consortium’s (ICGC) database [32]. These samples were pooled with whole genome mutation calls from The Cancer Genome Atlas (TCGA) melanoma cohort [21] called as described previously [10]. Population variants (dbSNP v138) and duplicate samples from the same patient were removed, resulting in a total of 221 tumors. Whole genome sequencing data from 5 *XPC* -/- cSCCs and matching peritumoral skin was obtained from Zheng *et al*., 2014 [5], and aligned with bwa (v0.7.12) [33] followed by mutation calling using VarScan 2 (v2.3) [34] and subtraction of population variants.

Gene annotations from GENCODE [35] v19 were used to define TSS positions, encompassing 20,149 and 13,307 uniquely mapped coding genes and lncRNAs, respectively, considering the 5ʹ-most annotated transcripts while disregarding non-coding isoforms for coding genes. Processed RNA-seq data was derived from Ashouri *et al*., 2016 [36]. Enhancer annotations were derived from ChromHMM segmentation (Core 15-state model, E6 and E7 regions, representing enhancers and genic enhancers, respectively) of epigenomic data from foreskin melanocytes (Roadmap celltype E059) [37].

### Culture and UV treatment of repair-deficient fibroblasts

XP12, GM16094, GM16095 and GM15893 cells were a kind gift from Dr. Isabella Muyleart, University of Gothenburg. Cells were grown in DMEM + 10% FCS + Penicillin/streptomycin (GIBCO). Cells were subjected to a single low dose UVB (20 J/m^2^) and left to recover for three weeks. DNA was extracted with Blood Mini kit (Qiagen).

### Ultrasensitive mutation analysis

To detect and quantify mutations we applied SiMSen-Seq (Simple, Multiplexed, PCR-based barcoding of DNA for Sensitive mutation detection using Sequencing) as described previously [17]. Sequencing was performed on an Illumina MiniSeq instrument in 150 bp single-end mode. Raw FastQ files were subsequently processed as described using Debarcer Version 0.3.1 (https://github.com/oicr-gsi/debarcer/tree/master-old). For each amplicon, sequence reads containing the barcode were grouped into barcode families. Barcode families with at least 10 reads, where all of the reads were identical (or ≥ 90% for families with >20 reads), were required to compute consensus reads. FastQ files were deposited in the Sequence Read Archive under BioProject ID SRP158874.

### Genome-wide mapping of cyclobutane pyrimidine dimers

A375 cells were grown in DMEM + 10% FCS + Penicillin/streptomycin (Gibco, Carlsbad, MA) and were treated with 1000 J/m^2^ UVC following DNA extraction and DNA from untreated cells was isolated as a control, both in duplicates. Additionally, naked DNA from untreated cells was irradiated with the same dose, to provide an acellular DNA control sample. DNA was extracted with the Blood mini kit (Qiagen, Hilden, Germany). Purified DNA (12 μg) was sheared to 400 bp with a Covaris S220 in microtubes using the standard 400 bp shearing protocol. CPD-seq was modified from Mao, Smerdon [26] to adapt it to Illumina sequencing methods using primers described previously in Clausen *et al*., 2015 [38] (S3 Table). Briefly, sheared DNA was size selected with SPRI select beads (1.2 vol) (Life Technologies, Carlsbad, CA) and the purified product (approx. 4 μg) subjected to NEBNext end repair and NEBNext dA-tailing modules (New England Biolabs (NEB), Ipswich, MA). ARC141/142 (8 μM) was then ligated to the sheared and repaired ends O/N with NEBNext Quick Ligation module. DNA was purified with 0.8 vol CleanPCR beads and treated with 40 units Terminal Transferase (TdT, NEB) and 0.1mM dideoxy ATP (Roche, Rotkreuz, Switzerland) for 2h at 37 °C. DNA was purified and incubated with 30 units T4 endonuclease V (NEB) at 37 °C for 2 h, followed by purification and treatment with 15 units APE1 (NEB) at 37 °C for 1.5 h. DNA was purified and treated with 1 unit rSAP (NEB) 37 °C 1 h followed by deactivation at 65 °C for 15 minutes. DNA was purified, denatured at 95 °C for 5 min, cooled on ice and ligated with the biotin-tagged ARC143/144 (0.25 μM) overnight at 16 °C with NEBNext quick ligation module. DNA fragments with the biotin tag were captured with 20 μl Streptavidin Dynabeads (Invitrogen, Waltham, MA) and the DNA strand without the biotin label was released with 2 x 40 μl 0.15 M NaOH and ethanol precipitated. This single-stranded DNA was resuspended in 14.9 μl H_2_O and used as the template to synthesize double-stranded products using ARC154 (0.25 μM) by incubating with Phusion High-Fidelity DNA Polymerase (Thermo Scientific, Waltham, MA) at 98 °C for 1 min, 58 °C for 30 s and 72 °C for 1 min. The now double-stranded library was purified and amplified for 15 cycles with ARC49 and ARC78-82 (0.3 μM each) to add Illumina barcodes and indexes. Two cellular UV-treated, two no-UV controls and one naked DNA control library were prepared, for a total of five libraries. The libraries were pooled with equal volumes of each of the libraries and sequenced using a NextSeq High Output kit (Illumina, San Diego, CA). The data has been deposited in GEO under accession GSE119249.

### CPD bioinformatics

FastQ files were aligned pairwise with Bowtie 2 version 2.3.1 [39] to hg19, using standard parameters. For the -UV control and +UV cellular DNA samples, replicates were merged with Picard MergeSamFiles version 2.18.7 (http://broadinstitute.github.io/picard). Duplicate reads were marked with Picard MarkDuplicates version 2.18.7 [40] with the parameter VALIDATION_STRINGENCY = LENIENT. Further analysis was performed in R with Bioconductor [41], where CPD positions were extracted as the two bases upstream and on the opposite strand of the first mate in each read pair, removing those that mapped outside of the chromosome boundaries. Only biologically possible CPDs detected at dipyrimidines sites were considered in the CPD counts and downstream analyses. Data from duplicate libraries were pooled to achieve higher coverage, since downstream results were in close agreement when considering these libraries individually. To simulate lower coverage libraries, the bam files were subsampled with samtools view version 0.1.19-44428cd [42] with the parameter -s at 0.25, 0.5 or 0.75, and the subsequent bam files were reanalyzed as described above.

For analyses of CPD formation patterns, C>T mutations and repair activity around TSSs, these regions were divided into 20 bp bins in which CPD counts or overlapping XR-seq reads were determined. XR-seq data from wild-type NHF1 skin fibroblasts was obtained from Hu *et al*., 2015 [43], and consisted of normalized read counts in 25 bp strand-specific bins. Background frequencies of dinucleotides and trinucleotides in hg19 were counted with EMBOSS’s fuzznuc [44], using the parameters -auto T -complement T. Expected mutations were calculated by randomly introducing the same number of mutations as observed in the window based on observed probabilities for C>T mutations at different trinucleotides estimated from the complete mutation dataset. Expected CPDs were calculated in the same way, maintaining the number of CPDs in the observed data, but based instead on genomic dinucleotide counts.

## Supporting information

S1 Fig Mutational burden and overall mutational signature for 221 melanomas. (**a**) Number of mutations in each sample, color-coded for pyrimidine-based nucleotide substitution. (**b**) Mutation frequency of each substitution type in different trinucleotide contexts, normalized for genomic trinucleotide background frequencies.
(PDF)

S2 Fig TTCCG-related recurrent mutations occur primarily in promoters rather than enhancers. (**a**) The location of recurrent mutations in melanoma relative to nearby annotated enhancers (analogous to Fig 1a which shows the position relative to nearby TSSs), based on chromHMM segmentation of Roadmap epigenomic data (E6 and E7 regions; genic enhancers and enhancers, respectively; median size 600 bp) from primary foreskin melanocytes. Although there is a perceived enrichment, most of the mutations are relatively far away from the annotated enhancers. (**b**) Same as panel **a** but after removal of promoter-proximal (within 1000 bp of a TSS) sites, revealing that the vast majority of recurrent mutations in panel **a** are in practice occurring in close proximity to transcription starts.
(PDF)

S3 Fig The nucleotide excision repair (NER) pathway, with mutated genes in the four repair-deficient cell lines (S1 Table) highlighted in red. (PDF)

S1 Table Recurrent TTCCG-related promoter mutations. 291 recurrent promoter mutations (+/- 500 bp from TSS), all mutated in at least 5/221 tumors and flanked by TTCCG elements (+/-10 bp sequence context).
(XLSX)

S2 Table Cell lines with DNA repair deficiencies and their verified homozygous mutations. Genotypes were verified by whole genome sequencing.
(PDF)

S3 Table Oligonucleotide sequences for CPD-seq. Illumina P5 and *P7* adapters are indicated underlined and italicized respectively, and **indexes** are shown in bold and underline. Oligo 5ʹ modifications are also indicated. All oligos were from Integrated DNA technologies (Coralville, IA). * indicates a phosphorothioate bond. /3Ammo/ indicates a 3' Amino Modifier. /5Phos/ indicates a 5´ phosphate. /5Biosg/ indicates a 5' Biotin.
(PDF)

S4 Table Numerical data underlying graphs. (XLSX)
