# Supplementary figures and images for: Elevated pyrimidine dimer formation at distinct genomic bases underlies promoter mutation hotspots in UV-exposed cancers
Source: PLoS Genet. 2018 Dec 26;14(12):e1007849. doi: 10.1371/journal.pgen.1007849 (PMC6329521; doi:10.1371/journal.pgen.1007849)

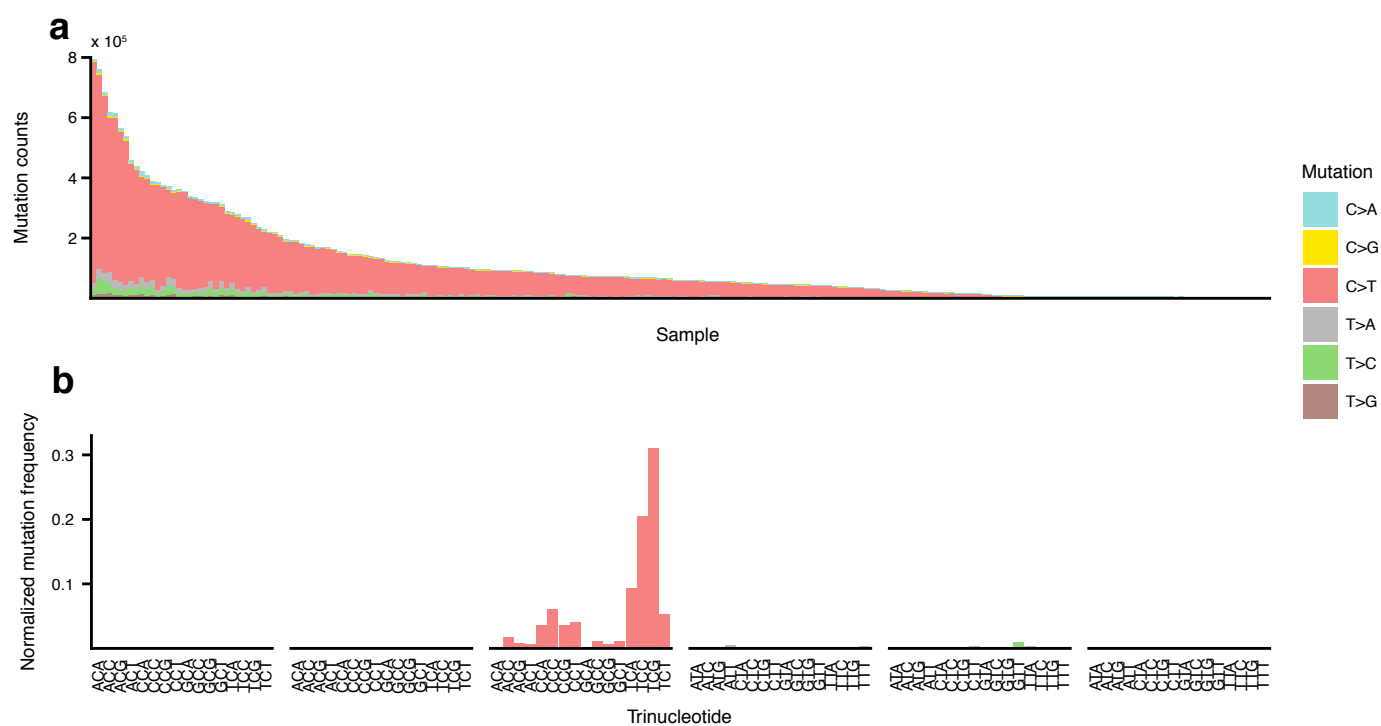

Supplement: S1 Fig — (a) Number of mutations in each sample, color-coded for pyrimidine-based nucleotide substitution. (b) Mutation frequency of each substitution type in different trinucleotide contexts, normalized for genomic trinucleotide background frequencies. (PDF) [file pgen.1007849.s001.pdf]

**a**

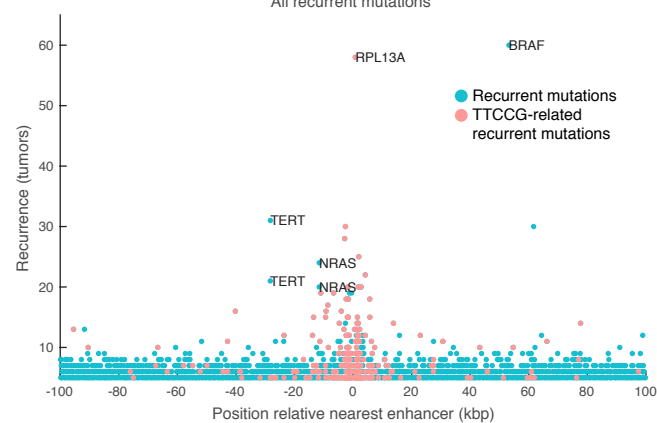

**b**

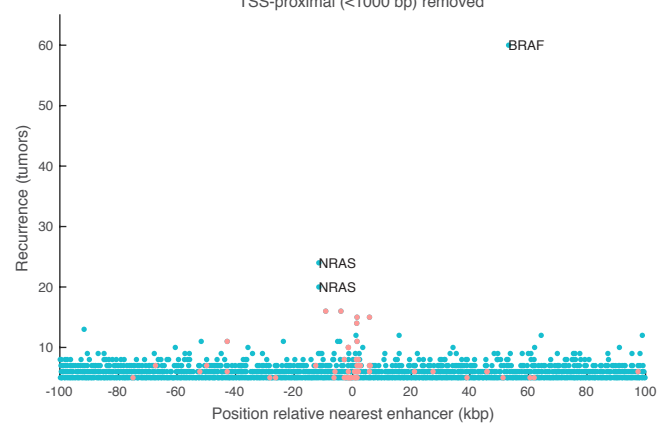

Supplement: S2 Fig — (a) The location of recurrent mutations in melanoma relative to nearby annotated enhancers (analogous to Fig 1a which shows the position relative to nearby TSSs), based on chromHMM segmentation of Roadmap epigenomic data (E6 and E7 regions; genic enhancers and enhancers, respectively; median size 600 bp) from primary foreskin melanocytes. Although there is a perceived enrichment, most of the mutations are relatively far away from the annotated enhancers. (b) Same as panel a but after removal of promoter-proximal (within 1000 bp of a TSS) sites, revealing that the vast majority of recurrent mutations in panel a are in practice occurring in close proximity to transcription starts. (PDF) [file pgen.1007849.s002.pdf]

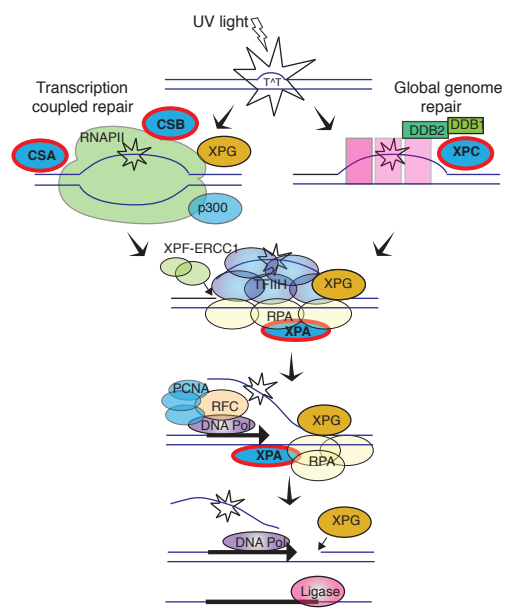

Supplement: S3 Fig — (PDF) [file pgen.1007849.s003.pdf]
