# Supplementary material for: Elevated pyrimidine dimer formation at distinct genomic bases underlies promoter mutation hotspots in UV-exposed cancers
Source: PLoS Genet. 2018 Dec 26;14(12):e1007849. doi: 10.1371/journal.pgen.1007849 (PMC6329521; doi:10.1371/journal.pgen.1007849)
Supplement: S2 Table — Genotypes were verified by whole genome sequencing. (PDF) [file pgen.1007849.s005.pdf]

Cell line	Gene	hg19 position	Protein substitution	Nucleotide substitution
XP12RO	*XPA*	Chr9:100447259	p.R207X	c.619 C>T
GM16094	*ERCC8* (*CSA*)	Chr5:60200621	p.Y145X	c.435 T>G
GM16095	*ERCC6* (*CSB*)	Chr10:50732467	p.K337X	c.1009 A>T
GM15983	*XPC*	Chr3:14199740	p.V548delTG	c.1643_1644delTG

S2 Table. Cell lines with DNA repair deficiencies and their verified homozygous mutations
