# Supplementary material for: Elevated pyrimidine dimer formation at distinct genomic bases underlies promoter mutation hotspots in UV-exposed cancers
Source: PLoS Genet. 2018 Dec 26;14(12):e1007849. doi: 10.1371/journal.pgen.1007849 (PMC6329521; doi:10.1371/journal.pgen.1007849)
Supplement: S3 Table — Illumina P5 and P7 adapters are indicated underlined and italicized respectively, and indexes are shown in bold and underline. Oligo 5ʹ modifications are also indicated. All oligos were from Integrated DNA technologies (Coralville, IA). * indicates a phosphorothioate bond. /3Ammo/ indicates a 3' Amino Modifier. /5Phos/ indicates a 5´ phosphate. /5Biosg/ indicates a 5' Biotin. (PDF) [file pgen.1007849.s006.pdf]

Primers	Sequence
ARC141/ 142	5′-GTGACTGGAGTTCAGACGTGTGCTCTTCCGATCT*T-3′
	5′-/5Phos/AGATCGGAAGAGCACACGTCTGAACTCCAGTCAC/3AmMO/-3′
ARC143/ 144	5′-/5Biosg/ACACTCTTTCCCTACACGACGCTCTTCCGATCTNNNNNN/3AmMO/-3′
	5′-/5Phos/AGATCGGAAGAGCGTCGTGTAGGGAAAGAGTGT/3AmMO/-3′
ARC154	5′-ACACTCTTTCCCTACACGACGCTCTTCCGATCT-3′
ARC49	5′-AATGATACGGCGACCACCGAGATCTACACTCTTTCCCTACACGACGCTCTTCCGATCT-3′
ARC78	5′-*CAAGCAGAAGACGGCATACGAGAT***CGTGAT**GTGACTGGAGTTCAGACGTGTGCTCTTCCGATCT-3′
ARC84	5′-*CAAGCAGAAGACGGCATACGAGAT***ACATCG**GTGACTGGAGTTCAGACGTGTGCTCTTCCGATCT-3′
ARC85	5′-*CAAGCAGAAGACGGCATACGAGAT***GCCTAA**GTGACTGGAGTTCAGACGTGTGCTCTTCCGATCT-3′
ARC86	5′-*CAAGCAGAAGACGGCATACGAGAT***TGGTCA**GTGACTGGAGTTCAGACGTGTGCTCTTCCGATCT-3′

Table S3. Oligonucleotide sequences for CPD-seq. Illumina P5 and *P7* adapters are indicated underlined and italicized respectively, and **indexes** are shown in bold and underline. Oligo 5’ modifications are also indicated. All oligos were from Integrated DNA technologies (Coralville, IA).
